# Graph Visualization: Alternative Models Inspired by Bioinformatics

**DOI:** 10.3390/s23073747

**Published:** 2023-04-04

**Authors:** Maxim Kolomeets, Vasily Desnitsky, Igor Kotenko, Andrey Chechulin

**Affiliations:** St. Petersburg Federal Research Center of the Russian Academy of Sciences, 199178 St. Petersburg, Russia; kolomeec@comsec.spb.ru (M.K.); ivkote@comsec.spb.ru (I.K.); chechulin@comsec.spb.ru (A.C.)

**Keywords:** data visualization, human–computer interaction, node-link diagrams, visualization models, review

## Abstract

Currently, the methods and means of human–machine interaction and visualization as its integral part are being increasingly developed. In various fields of scientific knowledge and technology, there is a need to find and select the most effective visualization models for various types of data, as well as to develop automation tools for the process of choosing the best visualization model for a specific case. There are many data visualization tools in various application fields, but at the same time, the main difficulty lies in presenting data of an interconnected (node-link) structure, i.e., networks. Typically, a lot of software means use graphs as the most straightforward and versatile models. To facilitate visual analysis, researchers are developing ways to arrange graph elements to make comparing, searching, and navigating data easier. However, in addition to graphs, there are many other visualization models that are less versatile but have the potential to expand the capabilities of the analyst and provide alternative solutions. In this work, we collected a variety of visualization models, which we call alternative models, to demonstrate how different concepts of information representation can be realized. We believe that adapting these models to improve the means of human–machine interaction will help analysts make significant progress in solving the problems researchers face when working with graphs.

## 1. Introduction

Both the natural and technical sciences, as well as even the humanities, are based on the analysis of data obtained during observations and experiments. In particular, the development of complex industrial and energy systems or the analysis of proteins, genes, chemical compounds, populations, metabolism, and various biological processes, as well as the social processes in social networks and other knowledge spheres, has long been associated with visualization [1,2]. Human–machine interaction presents a set of interfaces that allow a person to interact effectively with any computer device. Human–computer interaction comprises, first, ways in which a user transmits any information, commands, data on physical movements, user’s emotions, etc., to devices in digital form and, second, ways the user receives a response from devices in the form of text, visual, audio/video, and any other data. Therefore, as an element of human–machine interaction, visualization allows one to control the progress and analyze the results of experiments and provides multidimensional data with many attributes and relationships in simple graphical form.

In recent years, observing the development of high-performance experimental methods, the complexity of data analysis has increased many times. Despite this, most visual analytic tools use uniform visualization models, such as conventional 2D plots and histograms. In addition, as a rule, they use graphs to visualize the data of linked structures. However, in modern conditions of data explosion in many research fields, the types of visualization models used are not enough for effective visual analytics [3]. This problem is mainly expressed when using graph structures for large, complex, and not always interconnected data, when the search for specific components or attributes turns into a routine procedure, not much different from searching for data in a table [4,5,6,7].

A potential shift in solving this problem could be made by using alternative ideas for the graphical representation of data. This paper presents several ideas that were initially developed for bioinformatics tasks but can be applied to other research areas that use graph structures in visual analysis. These ideas are diverse, and we have tried to collect the ones being the most different from each other to show the possibilities and alternative ways in procedures of visual analysis of various structures. For developers of visualization tools, these ideas can be useful for increasing the variability in presentation methods both within individual tools and within the multiple-view paradigm [8,9].

This article discusses visualization models that are an alternative to common methods of graphical representation of information typically presented in the form of graphs and graph structures, which can be used to analyze data in various application areas. Most of the alternative models we observe in this paper are related to bioinformatics, as data visualization in this research area is well-developed and, therefore, extremely sophisticated. At the same time, it is possible to use the same concepts of graph representation in other research areas. Thus, the paper aims to observe visualization models from bioinformatics that are rare for other scientific domains and suitable for representing graphs with different topologies.

It is important to note that we do not address the paper to a specific research area and observe models on the level of data topology, highlighting that despite the use of the specific model in bioinformatics, it is also suitable for visual analytics in any analysis that uses similar graph structures.

The original methodology for selecting relevant visualization models used in this work considers the following factors:

1. Relevance of a visualization model and its focus on one or more application fields, which are characterized by specific data structures and logical relationships inside these structures;
2. Heterogeneity of the analyzed models as an opportunity to cover a significant number of visualization models that differ from each other, with aggregation of models based on the principles of their proximity and similarity, including hierarchical, planar, unstructured, temporal, multidimensional, and other models;
3. The ability to combine visualization models, including ones with several structures, to more fully display specific data characteristics, including the ability of an analyst to focus simultaneously on several types of information categories, such as overlaying a certain type of diagram on geomaps of the area;
4. The presence of practical confirmation of the applicability and usability of models in specific application cases.

The novelty of this work lies in the use of the original methodology for selecting visualization models proposed in the framework of this paper, including those being rare in practice, as well as in a comprehensive study of alternative ways of representing various data structures that are used in information technologies for visualization analysis tasks. In addition, the elements of the novelty include a high degree of heterogeneity of the data structure visualization models studied, which were not presented in previously published works in a comprehensive manner as visualization means and methods [10,11,12,13,14,15,16,17,18].

The contribution of this article is embodied in alternative visualization models, which can increase the variability of data visualization methods and thereby improve the possibilities of visual analysis. In addition, the article argues for the need for this study to increase the variability of the use of visualization models.

Overall, this paper aims to demonstrate the abilities of visualization models from bioinformatics so researchers can use them in other research areas if the same graph structures are present.

The rest of the article is organized as follows. Section 2 provides an overview of the data visualization field, including the challenges researchers face when working with graph models. Section 3 provides a list of specialized, rarely used visualization models and a classification of the data structures they use. Section 3.1 describes treemap, a visualization model widely used for displaying hierarchical data. Section 3.2 covers the Voronoi treemap model, a rendering model created as a solution to tree maps’ inherent aspect ratio and nesting problems. Section 3.3 analyzes the Voronoi map model, a visualization model that can be used as an alternative for representing planar graphs. Section 3.4 comprises an analysis of the chord diagram model as an example of representing a data structure with two different types of relationships. Section 3.5 analyzes the stacked chord diagram, a kind of visualization model that extends the chord diagram and can be used to analyze cognitive graphics tools. Section 3.6 covers the Voronoi diagram model, a model for visualizing unrelated objects that use a partition of the plane into sectors, depending on the position and parameters of the objects. Section 3.7 describes the trilinear coordinates model, a way to visualize three parameters relative to each other. Section 3.8 describes custom stacked models as examples of combining visualization models to display more data. Section 4 presents a discussion that comprises a comprehensive assessment of the possibilities, advantages, and disadvantages of the application of the specialized, rarely used visualization models. Finally, Section 5 concludes the article.

## 2. State of the Art

Graphs are the most common way to visualize various types of networks. Graphs are made up of at least two components: vertices, which represent objects, and edges, which represent connections between objects. In visualization, graphs are well studied; on their basis, many variations of building networks (hierarchical, radial, force, and others) were invented, and many software tools were developed that support working with graphs. At the same time, graphs are simple and intuitive, which leads to their wide distribution.

**Figure 1:**
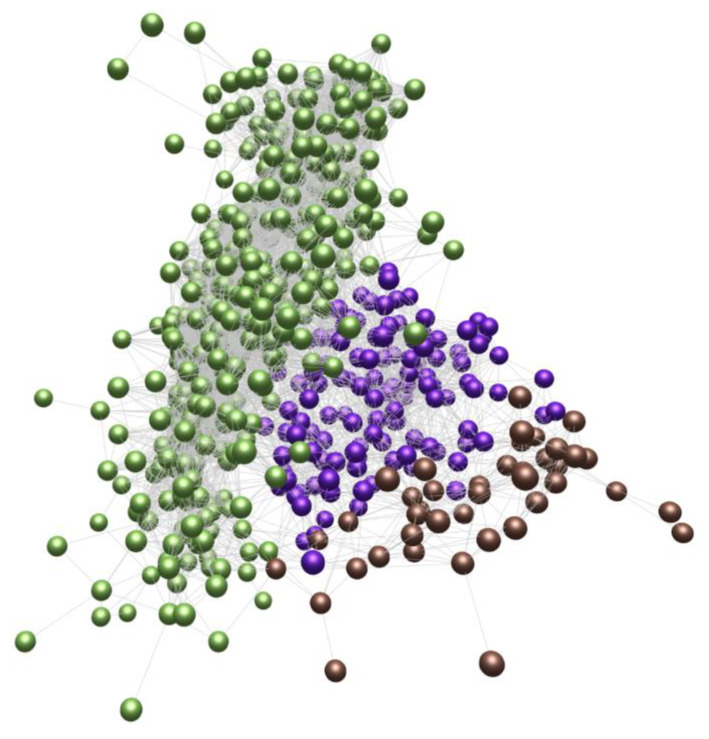
BioLayoutExpress3D example of analysis of the similarity (edge) between symptom profiles (vertex) [19].

Pavlopoulos et al. provide an overview and assessment of the software used for bioinformatics visualization [19]: Medusa [20], Cytoscape [21], BioLayoutExpress3D [22] (Figure 1), Osprey [23], ProViz [24], Ondex [25], PATIKA [26], and Pajek [22]. The authors of that review highlight the advantages and disadvantages of software tools and emphasize that the three main problems of network visualization are the increase in the amount of data, the problem of data heterogeneity, and the problem of representing multiple links between nodes. The software tools listed before cope with these problems to varying degrees, depending on the availability of certain functions. For example, when data heterogeneity is a major concern, integrative tools, such as Ondex [25] and Medusa [20], are suitable for analysis. In addition, for data with a large number of nodes, Cytoscape [21] and BioLayoutExpress3D [22] are suitable—these provide extensive navigation and scaling capabilities [27]. At the same time, the key visualization model that is used in the listed software is the graph model.

**Figure 2:**
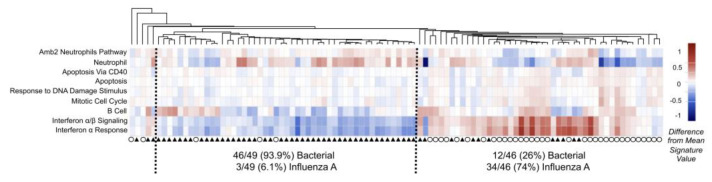
SaVanT visualization for expression data from patients with acute infections (influenza A—circle; bacterial pneumonia—triangle) [28].

**Figure 3:**
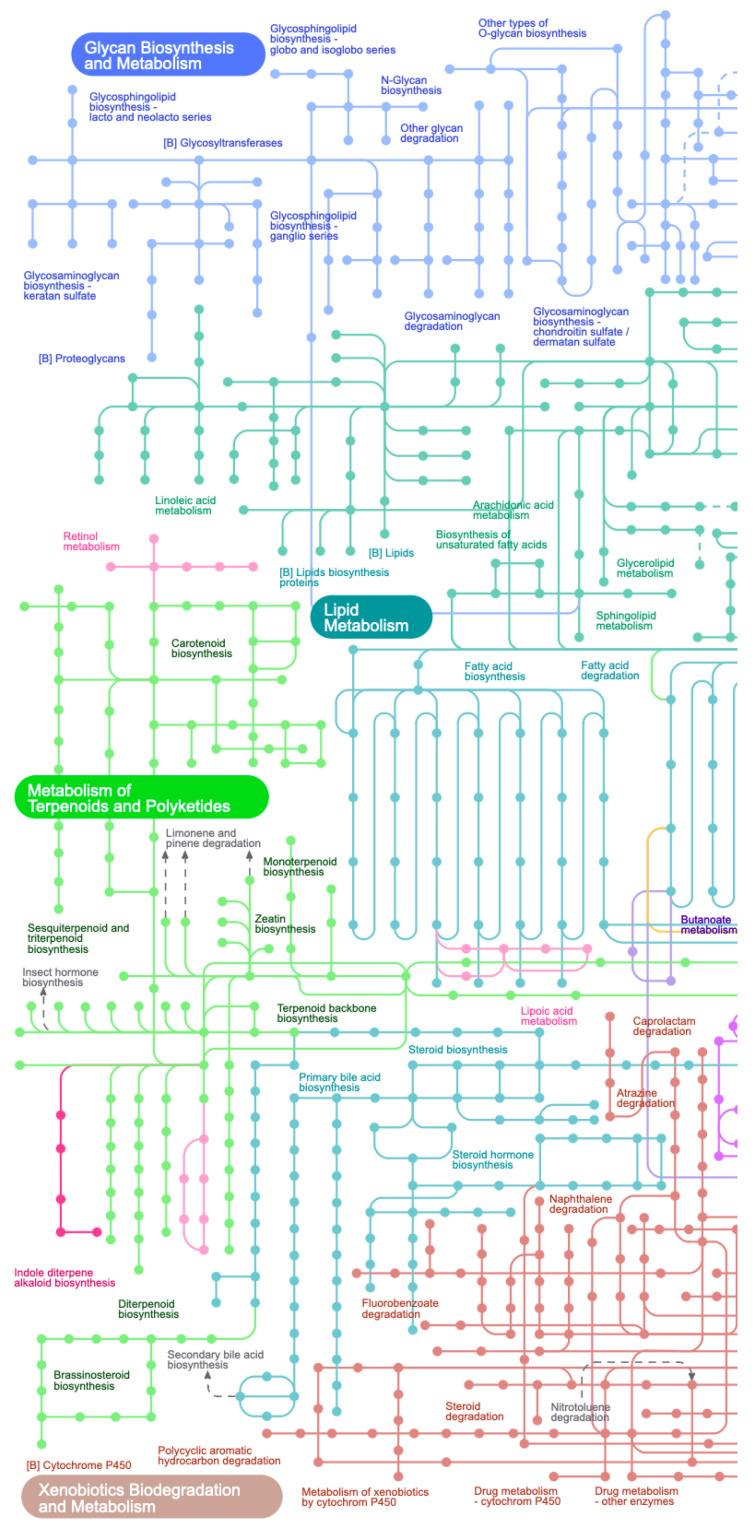
Visualization of metabolic pathways and profile data [28] made with iPath [29].

In addition, Gehlenborg et al. provide an analysis of the use of software (examples in Figure 2 and Figure 3) for visualizing bioinformatics data [28]. As in the previous work, the authors emphasize the problems of network analysis: problems of comparing networks with each other, issues of navigation and search with a large number of network nodes, problems of analyzing the dynamic properties of networks, etc. In the analyzed visualization software, graphs are also the key model. The authors conclude that in the future, improvements in the visual analysis will come from improved navigation methods that will help manage large or complex networks, increased web orientation that will simplify the interaction between researchers, and the use of technical tools, such as higher-resolution multi-touch screens. Interestingly, the authors also emphasize the need to go beyond the standard 2D view layouts and combine different layouts: 2D, 3D, and time.

As can be seen from the software used today, many data visualization tools have different functionalities for analyzing and arranging elements [30]. At the same time, the main difficulty in visual data analysis lies in the presentation of data of a related structure, i.e., networks. Therefore, major software tools use graphs as the most universal model that can be structured relatively easily and that support data visualization even with hundreds of thousands of nodes and no fewer links.

Obviously, networks and their elements can have many different parameters, attributes of objects, and topologies, which are not always easy to analyze on a graph. Therefore, graph theory began to play a major role in organizing storage, analysis, and visualization [31,32,33]. In the field of visualization, there are many works devoted to the methods of clustering graph elements, structuring methods, drawing techniques, building trees of various types, reducing data dimensions, etc. Researchers who deal with this problem agree that to simplify the visual analysis, and it is necessary to develop ways of arranging graph elements. However, in addition to graphs, there are many other alternative visualization models that are successfully applied in various fields. Of course, these models are not so universal, but in some cases, they have many advantages since they use relatively different ways of displaying nodes and links.

Concerning some application areas where graph data structures are common, for example, for the analysis of social networks, computer networks, transport flows, etc., the adaptation of alternative models from bioinformatics makes it possible to get partially rid of the problems inherent in graphs and can provide researchers with a potential alternative to graph models for analyzing the data in other research areas [34].

## 3. Alternative Models

In addition to graphs, many other models can also visualize networks. Currently, they are not widely used. This is partly because they can only visualize networks of a specific kind, while graphs do not have such restrictions. Another possible reason is that they are more complex in implementation and data preparation. However, in some cases, they can be a great alternative, presenting data more simply and understandably.

In this section, we observe such models from the perspective of data topology rather than a research area. Although these models are typical for bioinformatics, they are also suitable for other research areas as long as they use the same graph structures.

Networks can be characterized by the type of topology. The most straightforward classification is presented in Table 1.

Graph models are mainly designed to work with combined and unstructured networks. The obvious advantage of this approach is its versatility since with the help of models that support the visualization of unstructured networks, and it is possible to visualize planar, hierarchical, and unrelated data. However, many models are only for a specific type of topology and imply that the rendered data as a whole cannot be represented using another type of topology. In some cases, the use of such models can be more efficient than the use of graphs.

### 3.1. Table Representation

The table representation of graph data is the simplest method and is usually used for storage and graph processing. There are available several data storage formats, the most common of which are (1) the CSV format, which stores only edges as pairs of vertices or sets of edges and vertices as separate CSV files or stores relations in the form of an adjacency matrix, and (2) the JSON format, which stores edges and vertices in separate lists into a single file.

The table format is not a powerful technique, i.e., the complexity of the perception of patterns and object relations using the table format makes it useless for complex visual analysis and representation of graphs with a complex topology. Nevertheless, it is essential to mention it, as most graph data visualization techniques use this format as data input for their models.

### 3.2. Tree Maps

One of the most well-known ways to visualize hierarchical networks is treemaps [35,36,37,38,39]. In tree maps, each tree node is represented by a rectangle. Rectangles are nested in accordance with the topology of the tree: all ancestor nodes are located inside the descendant node. As a result, the treemap displays tree leaf rectangles located in the corresponding ancestor node rectangles, which are also located in the corresponding descendant node rectangles.

A feature of treemaps is that they display only the parameters of the tree’s leaves. In this case, the parameters of the nodes are formed based on child elements. Leaf parameters can be set using the size of the rectangles and color (including transparency, texture, saturation, and lightness). Thus, changing the size of a leaf rectangle entails changing the size of the entire hierarchy of rectangles in which it is nested. It follows from this that the size of the ancestor rectangle is always equal to the sum of the descendant rectangles.

**Figure 4:**
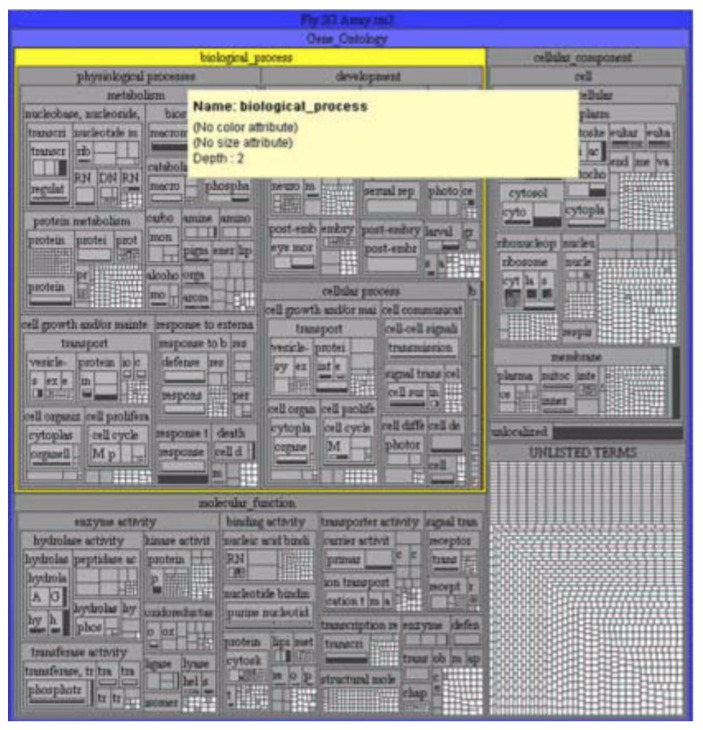
Gene ontology in the form of a treemap [35].

For example, in Figure 4, the selected biological process is highlighted in yellow, displaying more detailed information in other windows. The hierarchy of treemaps allows one to see all the data in their entirety and quickly navigate the structure.

### 3.3. Voronoi Treemaps

**Figure 5:**
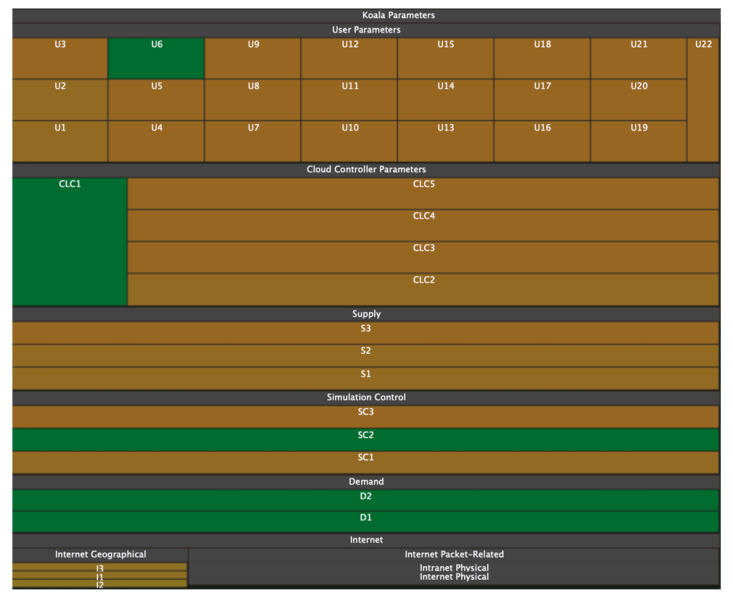
With a large scatter of parameter values, rectangles with a large width and height ratio may appear on treemaps [43].

**Figure 6:**
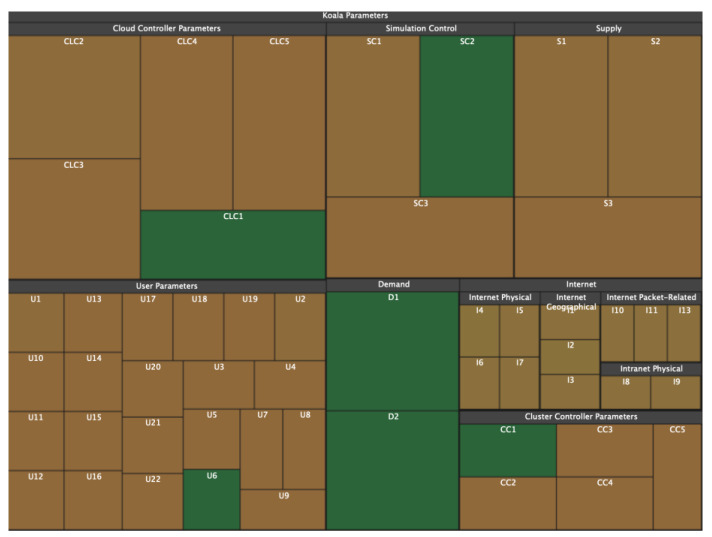
The tree shown in Figure 5 but redrawn using squarified treemaps [43]. It is noticeable that the desire for the equilateralness of rectangles gives rise to the complexity of determining hierarchical nesting.

The disadvantage of treemaps in terms of information perception (and, consequently, the speed and quality of analysis) is that with a large number of child nodes or with a large spread in the size of child nodes, rectangles with a large width-to-height ratio appear (Figure 5). This problem is solved by using ordered treemaps [40], squarified treemaps [41], or clustered treemaps [42]. The same tree in Figure 5 is redrawn using squarified treemaps [43] and presented in Figure 6. Another problem is the difficulty in defining nesting boundaries that result from using rectangular nodes.

**Figure 7:**
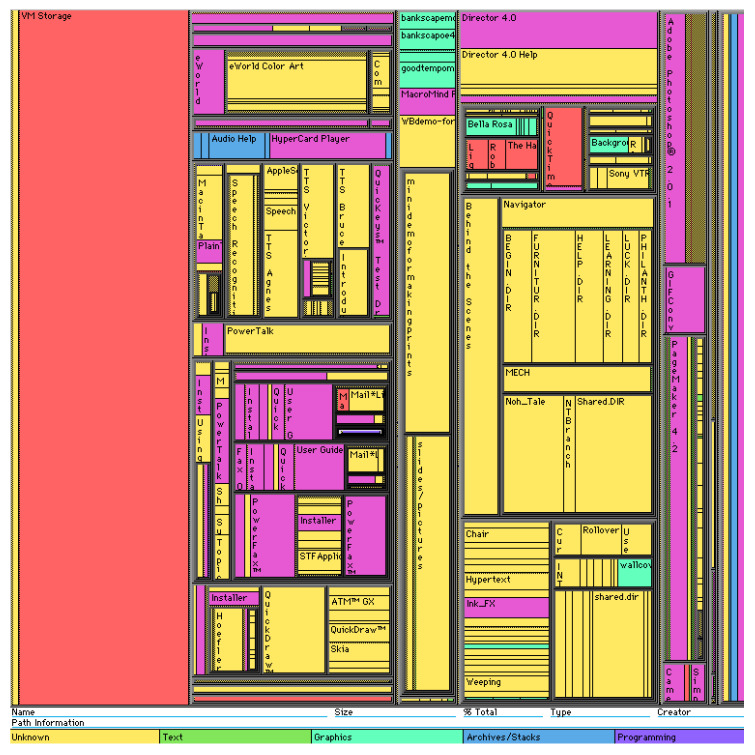
Hierarchical structure in the form of a map of trees [46].

**Figure 8:**
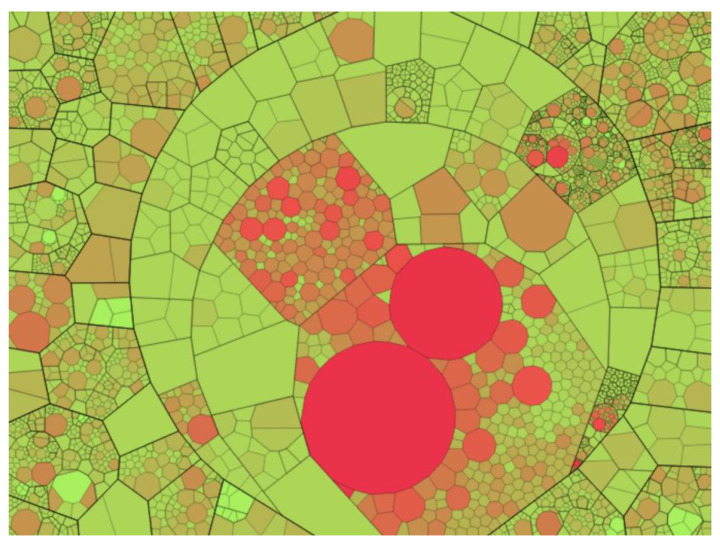
Hierarchical structure in the form of a Voronoi treemap [44].

Another visualization model that also expresses hierarchical networks and does not have the listed disadvantages is Voronoi treemaps [44]. Like tree maps (Figure 7), Voronoi treemaps (Figure 8) consist of nested regions that can be specified in color, size, and transparency; however, tree nodes are presented not by means of rectangles but by polygons. The use of polygons allows one to effectively distinguish different objects from each other and thereby level out the disadvantages of information perception inherent in treemaps [45].

### 3.4. Voronoi Maps

Another problem that is inherent in both maps of trees and Voronoi maps of trees is expressed in the hierarchy itself. First, the area of the ancestor polygon is equal to the sum of the descendant rectangles. As a consequence, we can only visualize the metrics of the leaves, and the metrics of the higher nodes must be expressed as the sum of the lower nodes in the hierarchy. Second, by definition, tree maps and Voronoi treemaps can only display hierarchical structures. Third, by changing the size and color of the polygons, tree maps and Voronoi treemaps can visualize the parameters of the nodes but cannot visualize the parameters of connections between the nodes. In these visualization models, a connection appears exclusively in the form of a nesting of polygons and uniquely corresponds to the topology.

**Figure 9:**
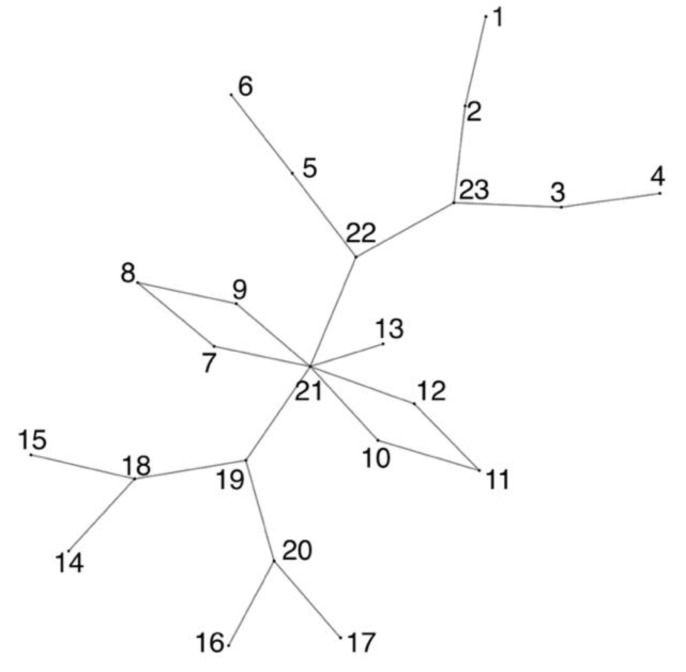
Planar graph.

**Figure 10:**
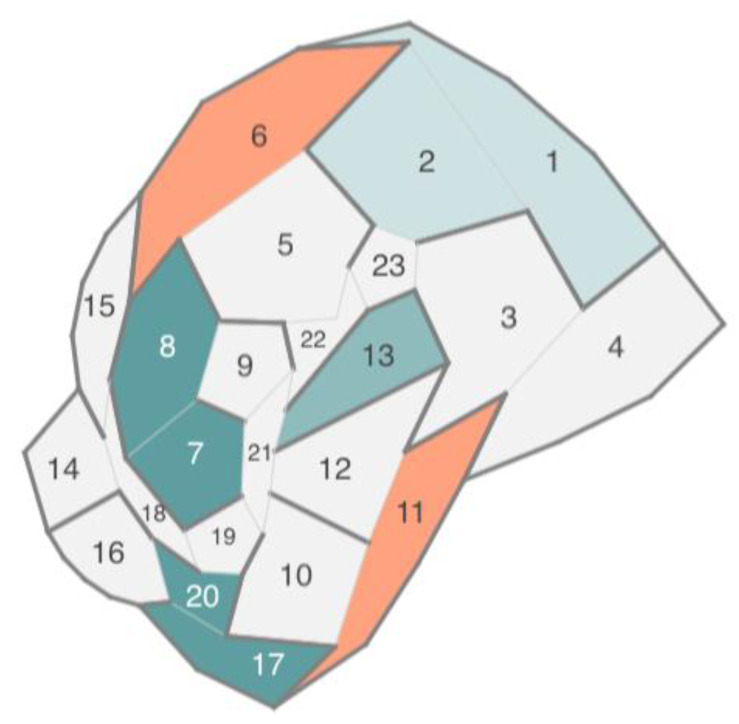
Voronoi map based on a planar graph.

Thus, Voronoi treemaps have been further developed in the form of Voronoi maps [47], which display the topology based on the ratio of polygons rather than their nesting. Voronoi maps can display networks with a planar topology, in which a polygon represents the network node without intersections, and the connections between the nodes are represented by the contact of polygons with edges. In such a structure, separators can also appear, i.e., edges that, on the contrary, separate rather than connect nodes. An example of a Voronoi map and the graph on whose basis it was built are shown in Figure 9 and Figure 10.

Voronoi maps eliminate the listed shortcomings of tree maps and Voronoi treemaps: They can display node parameters independent of each other using the color, size, and transparency of the polygon; they can display not only hierarchical but also planar networks, and they can display the parameters of connections between network nodes by using the color, transparency, and thickness of adjoining edges.

A good analogy for this approach is a labyrinth (maze). Each cell of the map (node) is a maze room, some cell edges (connections between nodes) are doors, and other cell separator edges (no connection between nodes) are walls. The topology of the structure is perceived as an ability to move between rooms, while the parameters of nodes and their connections are perceived by various indicators of rooms and doors (colors, sizes, position).

The disadvantages of the Voronoi map are the ability to build a map for networks with planar topology only and the difficulty in resizing the polygons of the Voronoi map to display node parameters as a polygon size.

As a result, the disadvantage of this type of diagram is that when choosing them, first, the researcher has to determine the scale (in this case, the number of axes) of the data, which causes possible inconvenience when further manipulating the model images.

### 3.5. Chord Diagrams

**Figure 11:**
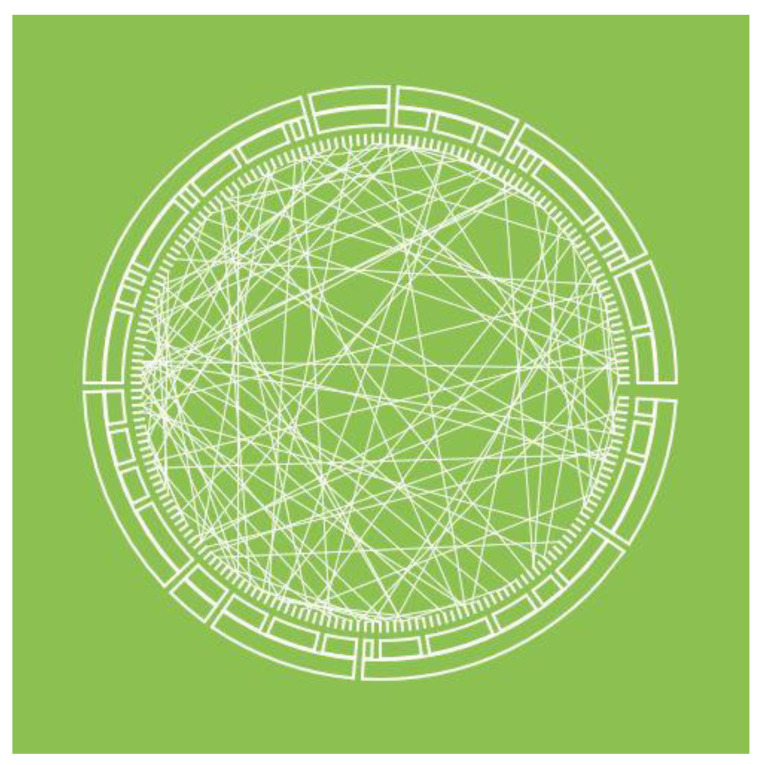
Chord diagram with straight edges. The outer shell consists of three rings, displaying a hierarchical topology. The inner shell consists of an unconnected graph.

**Figure 12:**
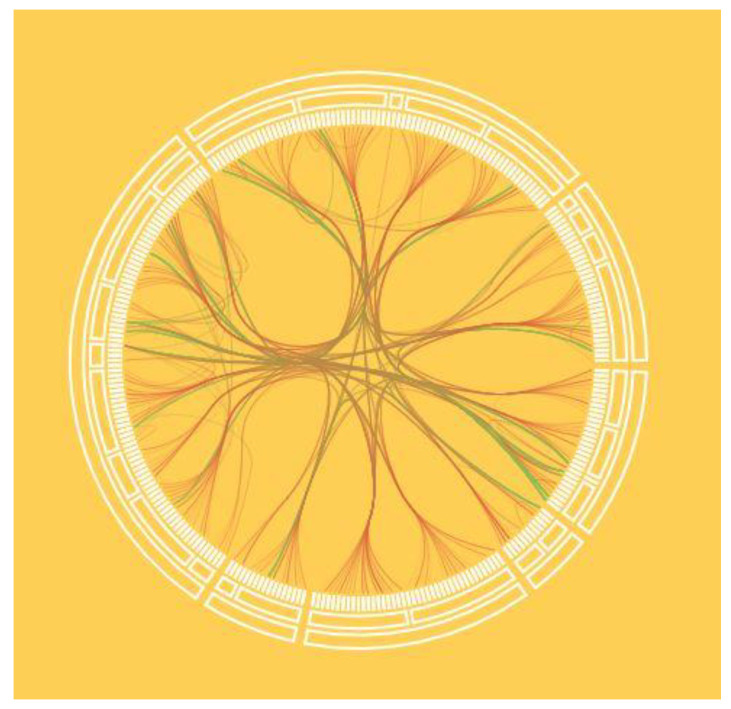
Chord diagram with seventh-order Bezier curves. Bezier curves allow one to “wisp” the connections of an unconnected graph and improve the readability of the image.

There are networks with two types of links between nodes: hierarchical links and unstructured ones. They can be presented as a special case of the chord diagram (Figure 11 and Figure 12) [48,49,50,51]. In this approach, the object hierarchy is displayed as an inverted radial graph. Inside it, in the first ring, are the leaves of the tree. On subsequent rings, the ancestor nodes of the elements of the previous rings are located. The connection between the nodes is denoted as the presence of common x-coordinates in the radial reference system.

Unstructured links are displayed as a graph located inside the rings. The graph edges can be displayed both as straight lines (Figure 11) and as N-order Bezier curves (Figure 12). In this case, the order of the curves depends on the number of degrees of the hierarchy. Radial tree node parameters can be displayed as colors, and unstructured graph link parameters as the thickness, color, and transparency of an edge or curve.

### 3.6. Stacked Chord Diagrams

The stacked chord chart is a modified version of the chord chart and is used to display streaming or temporal data, with the ability to use cognitive graphics for visual analytics.

**Figure 13:**
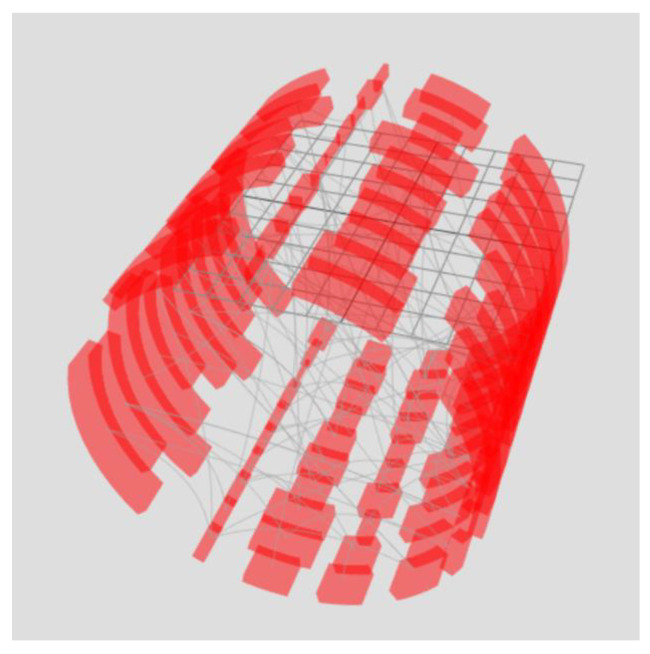
Stacked chord diagram. Each chart represents one-time slice. The arrangement of arc elements of the ring one after another provides information about the change in the parameter over time by analogy with a flowchart.

A stacked chord diagram is a torus that consists of rings, and each ring is located along the z-axis and displays a specific state at the corresponding moment in time. Rings consist of arc nodes. Node options can be displayed as the arc length, arc thickness, arc color, or arc transparency. Thus, the rings, located one after another, reflect the dynamics of the parameters change over time (Figure 13—only one parameter is used in the figure in the form of the arc length). Analogous to the chord diagram, inside the rings, there is an unstructured graph that connects the elements of the rings.

**Figure 14:**
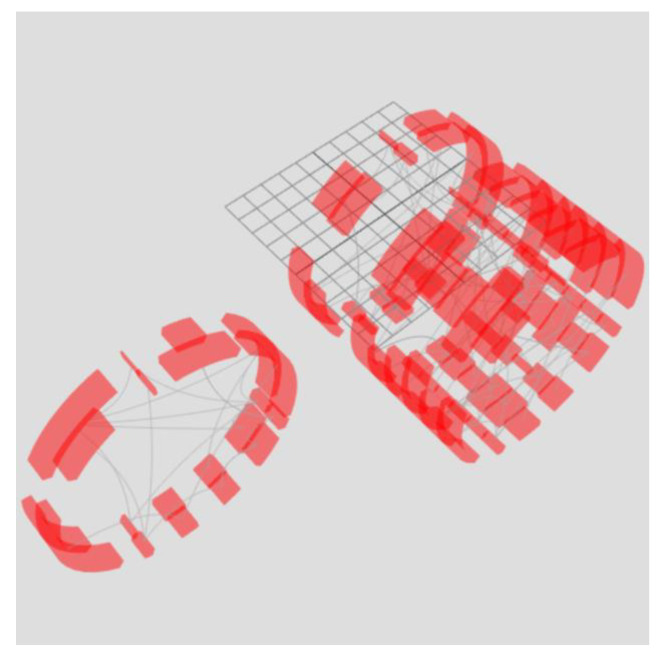
Moving part of the rings outside the torus allows one to select part of the time slices, forming a second filtered torus. At the same time, filtering occurs not by manipulating data but by manipulating graphics as if they were real physical objects.

**Figure 15:**
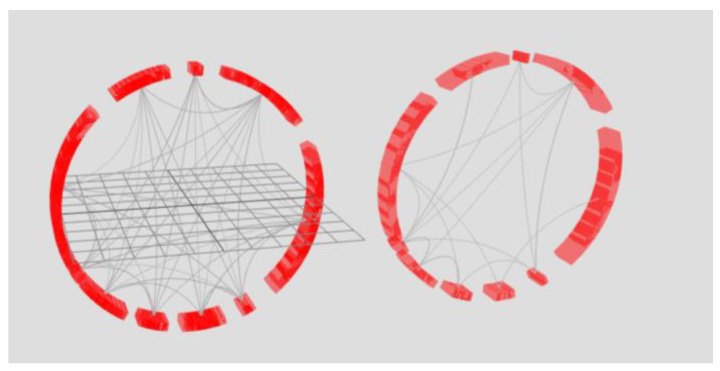
Combination of rings of tori.

The power of cognitive graphics manifests itself as filtering the data set through graphical interaction rather than making changes to the displayed data set. For example, the exclusion of a time slice from the sample or the formation of a new set is possible by moving the ring outside the torus (Figure 14). Another example is the alignment of all rings (Figure 15). As a result of superposition, element connections for all periods are displayed—arc lengths display the maximum value of the arc element parameter for the entire period of the analyzed time, and edge opacity shows the probability distribution of the minimum parameter value. To display the probability distribution of the minimum value, the initial transparency of the edges must be equal to $\frac{100\%}{\mathrm{Number}\;\mathrm{of}\;\mathrm{rings}}$.

On the left side of Figure 15, eight rings of the right torus in Figure 14 are combined. On the right side, there are two rings of the left torus in Figure 14. Superimposing the rings on top of each other allows one to obtain information about the bonds for the periods represented in the tori. The key feature is that the data are not processed, i.e., the analysis occurs through graphical manipulations as if over a physical object using cognitive graphics tools.

### 3.7. Voronoi Diagrams

**Figure 16:**
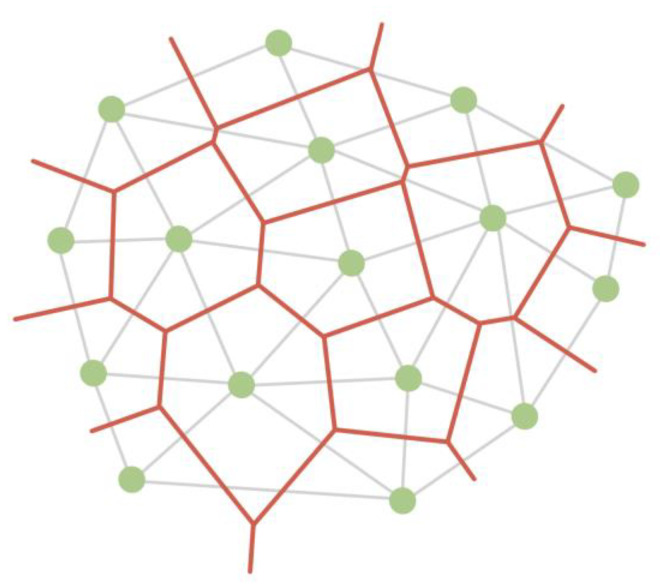
A Voronoi diagram (red) partitions a plane into cells based on triangulation (grey edges) of centroids (green vertices).

A Voronoi diagram can be used to visualize compound data structures, such as ones describing proteins, atoms, or amino acid residues. This model is built on the basis of points (centroids) and divides the plane into polygons, which are called Voronoi cells. Each centroid corresponds to a Voronoi cell. In the classical Voronoi diagram, the cells have the following mathematical meaning: any point of the Voronoi cell is closest to the centroid based on which the cell was built (Figure 16). Each cell can be considered a zone of influence of an atom or other object, played by a point [52,53,54,55].

**Figure 17:**
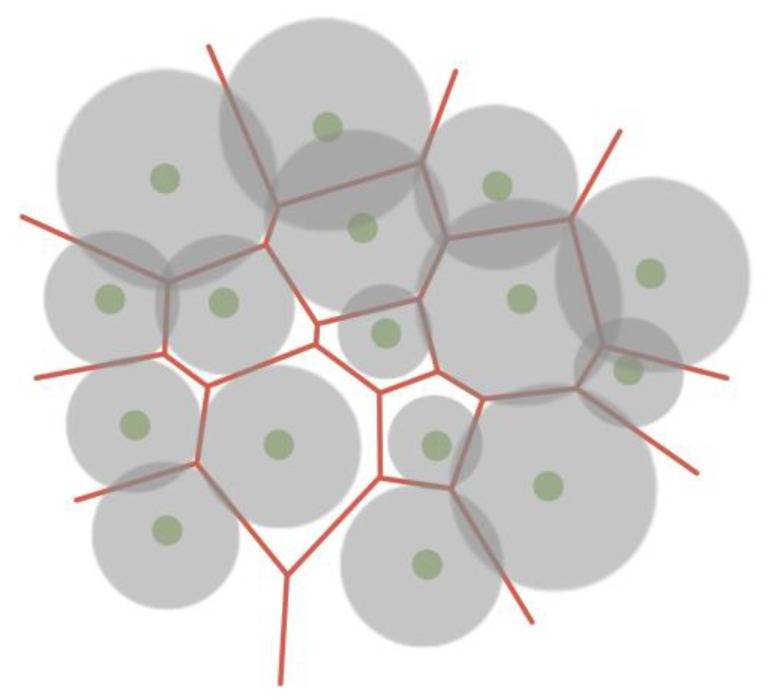
Voronoi force diagram, taking into account the weights of centroids, where the parameter of the atom determines the weight.

**Figure 18:**
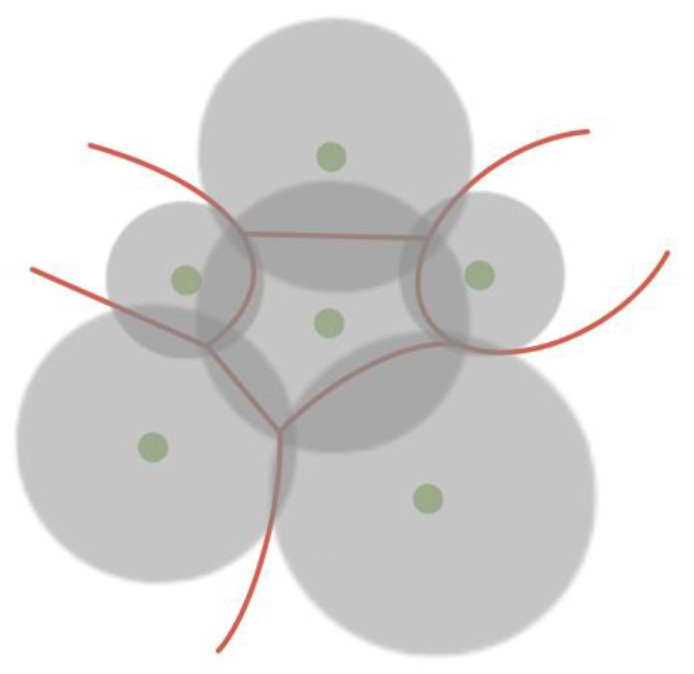
Voronoi diagram in which the distance to the separating edge is related to the weight of the centroid. This approach makes it possible to obtain curved edges of Voronoi cells.

**Figure 19:**
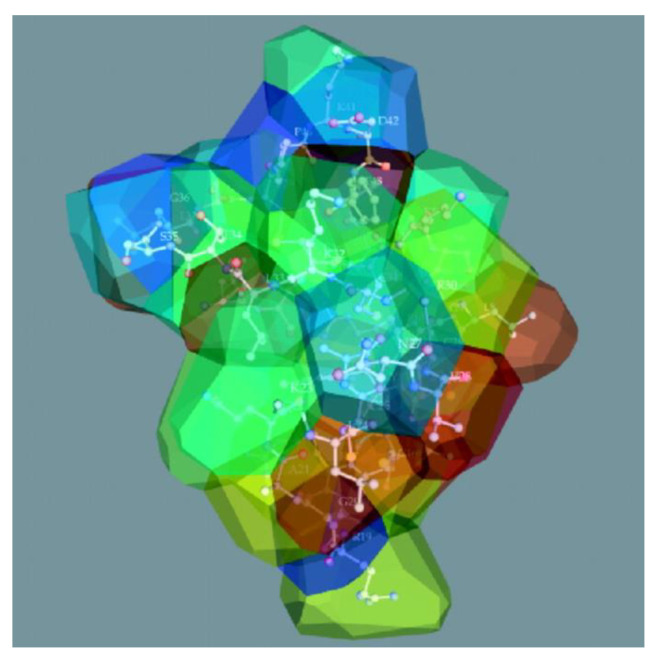
The structure of beta-purothionin in the form of a 3D Voronoi diagram, which was built taking into account weighting factors [59].

At the same time, there are various algorithms for constructing a Voronoi diagram, which can operate with the weight of the centroid, thereby taking into account the properties of atoms based on which the partition is built (Figure 17, Figure 18 and Figure 19) [56,57,58].

In addition to splitting the plane, there are algorithms for constructing a 3D model of the Voronoi diagram by splitting the space into polyhedra, which can also operate with the weight of the centroid (Figure 19) [59,60,61].

### 3.8. Trilinear Coordinate Model

A trilinear coordinate model can be used to display relative data. An example of trilinear coordinates is the United States Department of Agriculture’s soil texture triangle, which is used to define soil types. Robert Bruce Whitaker developed this idea by proposing the use of the trilinear coordinate model [62,63] to display any three or two object parameters relative to each other.

**Figure 20:**
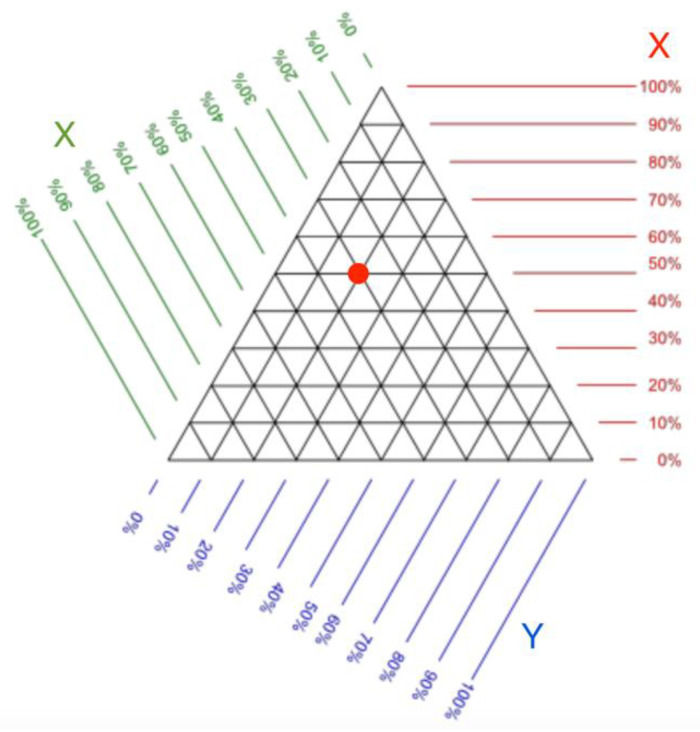
Trilinear coordinate pattern. It can be used to determine the value of the parameters of an object located on a triangle. The red dot denotes an example of data in trilinear coordinates [62].

The trilinear coordinate model is a triangle whose sides represent the object’s parameters. The object is depicted as a point located in a trilinear coordinate system. Figure 20 shows a template where the object (red dot) has x, y, and z values of 50%, 20%, and 30%, respectively.

**Figure 21:**
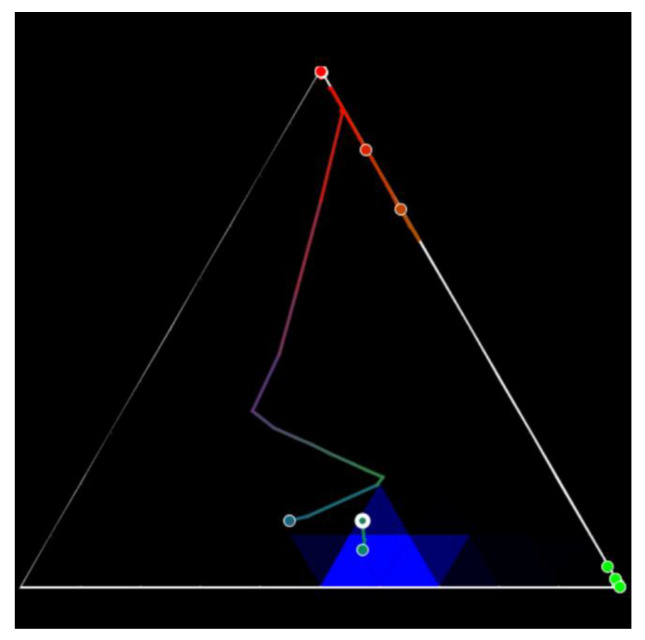
Trilinear coordinates illustrate the dynamics of objects. A change in parameters is represented by a trajectory, and the area of typical parameters for the object is highlighted in blue. The colors of the trajectory from warm (red) to cold (blue) indicate the rate of change in the ratios of the three coordinates used [62].

The trilinear coordinate model can be used to detect deviations from the typical values of an object over time. An example of such use is shown in Figure 21. Figure 21 exposes several objects and their trajectories that are formed when changing the ratio of three parameters. Trajectories are highlighted with a color that indicates the rate at which the ratio changes. In this case, it is possible to single out areas in which the presence is typical for the object. Areas can be identified based on historical data on the range of values in which the object parameters have been the longest. In Figure 21, one of these areas is highlighted in blue.

### 3.9. Custom Stacked Models

Separately, visualization models can be distinguished. These are formed by linking other models [64]. Such models rely on the structuring of a data set, highlighting the layers of data into a hierarchy. For example, the first layer can be a network and its parameters, and the second one can be an object and its parameters. In this case, each individual layer is represented by a different model. Such models can be diverse and, in fact are a combination of different models [65].

This approach can be used when the visualization model does not allow exposing many parameters. For example, in the case of visualizing a network using a graph, the object parameters can be represented as the graph vertex size and vertex color. It is also possible to display object parameters in the form of vertex transparency or vertex texturing. However, it is obvious that this way of representation will be difficult to perceive.

**Figure 22:**
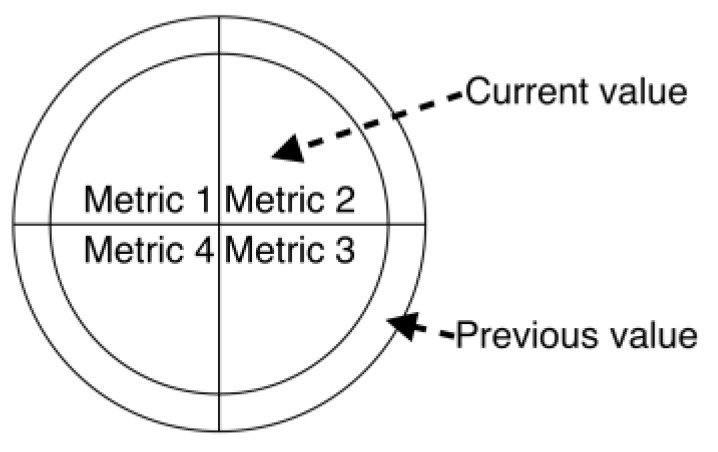
An example of a glyph with four kinds of parameters. The inner part of the glyph displays the current values, while the outer part shows the previous ones.

**Figure 23:**
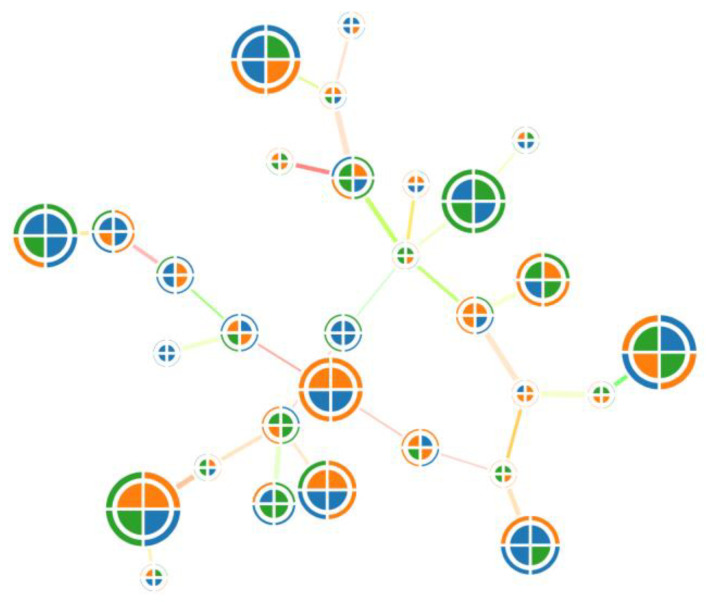
An example of a graph combined with glyphs. Due to glyphs, it becomes possible to use more object parameters.

Using the example of a graph, stacked models imply that a vertex can be changed to another model that can accommodate more parameters, such as a glyph (Figure 22). Glyphs are made up of several parts, and each part presents a parameter with a color. Glyphs can also have layers that present the typical or previous setting value. An example of a graph combined with glyphs is shown in Figure 23.

### 3.10. Virtual and Augmented Reality

The use of virtual reality (VR) and augmented reality (AR) for data visualization is promising [66,67,68,69,70,71]. Graphic models in VR and AR are not limited by screen size, so they can accommodate a huge amount of data. Furthermore, in VR and AR, one can interact with data as with real physical objects [72,73,74]. This allows one to intuitively compare and arrange data. In general, the use of VR and AR allows coping with several disadvantages inherent in traditional approaches to visualization.

Virtual reality and augmented reality are relatively new areas since for a long time, VR and AR devices were not available due to their rather narrow professional orientation (for air pilots, race car drivers, etc.) or high cost, which significantly limited their widespread use [75,76,77]. In 2012, the first affordable virtual reality device, the OculusRift, was introduced. Later, similar affordable devices appeared, including Samsung Gear VR (2014), HTC Vive (2015), Sony PlayStation VR (2016), Google Daydream (2016), Oculus Quest (2021), and HTC Vive Flow (2022). The devices themselves are positioned by developers as gaming devices or devices for learning.

Augmented reality devices are a separate segment among virtual reality devices. Unlike virtual reality, in augmented reality, the image is superimposed upon real physical objects. On the one hand, this allows one to interact with both the virtual and the physical world; on the other hand, it requires more sensors that are necessary to determine the location of not only the user but also the objects around this user. The following augmented reality devices are currently available: GoogleGlass, MetaVision, and Microsoft HoloLens. Unlike virtual reality devices, augmented reality devices are positioned as aids for 3D model designers, architects, and other professional activities [78,79].

It should be noted that there is no single concept of how to develop data visualization in VR and AR. Despite this, solutions for particular data visualization tasks in VR and AR are already used by various companies [80,81].

1. A group of scientists from South Korea and the United States investigated ways to visualize graph structures in virtual reality on various types of spheres [82].
2. Ana Becker (data journalist of The Wall Street Journal) visualized the history of NASDAQ exchange quotes in virtual reality [83], which shows the possibilities of using VR in education and journalism.
3. Michal Koutek and Fritz Post developed the MolDRIVE visualization system, which makes it possible to visualize and control experiments in the field of molecular dynamics [84].
4. Bob Levy presented the VirtualCove project, which visualizes stock indices in augmented reality [85].
5. E-Semble develops emergency simulation programs to train qualified personnel.
6. Brown University (Providence, RI, USA) uses virtual reality for various scientific experiments and teaching in psychology, surgery, geology, bioengineering, and other fields [86].
7. At the Engenharia Nuclear Institute (Rio de Janeiro, Brazil), the possibilities of using virtual reality to ensure the functioning of nuclear power facilities are being explored [87].

Research on visualization through virtual and augmented reality is currently undergoing rapid growth. However, significant fundamental studies on the design of visualization systems in VR and AR have not been conducted. However, to date, the necessary basis has been formed for developing data visualization systems using virtual and augmented reality [88,89].

## 4. Discussion

In this section, we discuss the advantages and disadvantages of the observed models. It is necessary to mention that the main functionalities and resources strongly depend on the implementation of the specific model. Therefore, it is impossible to compare speed for rendering, the maximum number of supported metrics, perception complexity [10,90], and similar criteria that are usually essential for model selection on the application level. However, we highlight that the supported topology type is the main limit for applicability, as models can visualize only a specific class of graph topology. For that reason, we propose model comparison from the following perspectives: (1) the ability of a model to visualize the complex topology of a graph and (2) how different research areas can use these models in the context of the structures that are most common for this scientific domain.

Most data visualization tools in various subject fields use graphs to visualize related data and graphs or histograms for unrelated data [91,92]. However, visualization models, which are alternatives to them, have yet to be widely used. There are several reasons this happens. First, in trivial cases, simple models are sufficient for researchers, which are well studied and have many implementations that support various file formats. Second, the models presented in the article are not universal—they can only visualize specific structures (Table 2). At the same time, researchers use graphs in any case, albeit with varying degrees of efficiency. Third, most of the presented alternative models are challenging to implement, leading to their occasional use in existing software.

However, alternative models turn out to be more effective for specific tasks in the field of human–computer interaction or when the data structure is known in advance [93,94]. One can classify examples presented in the paper according to the types of data structure topologies: (a) tree maps and Voronoi treemaps are hierarchical data, (b) Voronoi maps represent planar data, (c) chord diagrams and stacked chord diagrams are hierarchical data together with planar data, (d) Voronoi diagrams and trilinear coordinates are unrelated data, and (e) custom stacked models represent stacked data types, depending on the implementation.

It is evident that the higher the topology number from Table 1 the model supports, the more universal it is. For example, Voronoi maps can visualize planar data, including hierarchical and unrelated data. In addition, graphs can visualize the list of topologies less efficiently than specialized ones. Based on the literature analysis, Table 3 provides examples of comparisons of some regarded generalized application fields in the context of using both common visualization models and alternative ones. This confirms the applicability of the alternative visualization models studied in the article in conducting visual analysis of data of different natures.

In this article, we presented visualization models most different from each other to demonstrate how various concepts of information presentation can be used [95,96]. These models can be extended by their use in 3D (e.g., the Voronoi diagram in Figure 16 and Figure 19) or by combining them with other models [97]. We believe that adapting existing models to the needs of different subject fields and creating new visualization models is another way to solve existing problems.

## 5. Conclusions

In the article, we considered visualization models as an alternative to graphs as a component of human–machine interaction, inspired by the visualization approaches used in bioinformatics. Despite their low prevalence, it has been shown that they can be an alternative to the established methods of presenting data in various application areas, such as transport systems [34,98], water supply management systems [99], critical infrastructures [100], mobile payment systems [101], social networks [102], and digital forensics [103]. The specificity of each model was shown, and the models leveled out the shortcomings in each other. In addition, for the considered models, a classification was given according to the type of topology of the supported data structures, so the observed models can be used in applications that differ from bioinformatics but with the same graph structures. The advantages and disadvantages of using alternative visualization models compared to graphs were provided. Finally, recommendations were given on the use of the presented visualization models with various data structures.

The research in this field is planned to be continued. In particular, it is supposed to explore the possibilities of the aforementioned visualization models for solving many specific problems of one class, including those in biology, soil science, materials science, and other areas. In addition, it is necessary to qualitatively evaluate the efficiency of use and the speed of implementations of alternative models on large data sets [104,105]. It is also planned that ways to build combined models based on existing ones will be investigated in order to increase their universality [106,107,108].

## Figures and Tables

**Table 1:** Characteristics of network topologies.

No.	Topology type	Description
1	Unrelated data	A data set that does not present a network (a set of features with no links between them)
2	Hierarchical networks	Planar networks that can be represented as a tree
3	Planar networks	Networks for which planarity can be proved and for which a planar image in the plane can be found in polynomial time
4	Unstructured networks	Networks for which it is impossible to single out a single-valued structure or whose structure is impossible to determine due to the large amount of data that cannot be analyzed in polynomial time
5	Combined networks	Networks that have several topology levels (a set of objects with links of different types), while different topologies do not have to be of the same type

**Table 2:** Correspondence between a network topology and alternative visualization models.

No.	Topology Type	Visualization Model
1	Unrelated data	Voronoi diagrams, trilinear coordinates, heat maps
2	Hierarchical networks	Treemaps, Voronoi treemaps
3	Planar networks	Voronoi maps
4	Unstructured networks	Graphs, matrices
5	Combined networks	Chord diagrams, stacked models

**Table 3:** Usage of visualization models.

Examples of Application Fields	Common Visualization Models (Topology Complexity Is Presented in Parentheses)	Alternative Visualization Models
Bioinformatics	Heat maps (1), graphs (4), planar networks (3), matrices (4), etc.	Treemaps (2), Voronoi diagrams (1), VR (5)
Sociology, economy	Hierarchical networks (2), unrelated data (1), matrices (4), etc.	Voronoi treemaps (2), VR (5), AR (5)
Agrosphere and soil science, geology	Geomaps (1), planar networks (3), combined networks (5), etc.	Trilinear coordinate model (1), Voronoi maps (3), VR (5)
Medicine and neuroscience	Graph of a function (4), trees (4), etc.	Chord diagrams (5)
Cyber-security	Trees (2), matrices (4), scatter plots (1), parallel coordinates (1), heat maps (1), etc.	Treemaps (2), Voronoi maps (3), stacked models (5)

## Data Availability

Data sharing not applicable.
